# Inflammatory and lipid regulation by cholinergic activity in epicardial stromal cells from patients who underwent open‐heart surgery

**DOI:** 10.1111/jcmm.15727

**Published:** 2020-08-07

**Authors:** Marinela Couselo‐Seijas, José N. Lopez‐Canoa, Ángel L. Fernandez, Laila González‐Melchor, Luisa M. Seoane, Darío Duran‐Muñoz, Adriana Rozados‐Luis, José Ramón González‐Juanatey, Moisés Rodríguez‐Mañero, Sonia Eiras

**Affiliations:** ^1^ Translational Cardiology group Health Research Institute Santiago de Compostela Spain; ^2^ University of Santiago de Compostela Santiago de Compostela Spain; ^3^ Cardiovascular Department University Hospital of Santiago de Compostela Santiago de Compostela Spain; ^4^ Heart Surgery Department University Hospital of Santiago de Compostela Santiago de Compostela Spain; ^5^ Cardiology Group Health Research Institute Santiago de Compostela Spain; ^6^ CIBERCV Madrid Spain; ^7^ Endocrine Physiopathology Group Health Research Institute Santiago de Compostela Spain; ^8^ CIBERobn Madrid Spain

**Keywords:** atrial fibrillation, epicardial fat, parasympathetic dysfunction

## Abstract

The modulation of acetylcholine (ACh) release by botulinum toxin injection into epicardial fat diminishes atrial fibrillation (AF) recurrence. These results suggest an interaction between autonomic imbalance and epicardial fat as risk factors of AF. Our aim was to study the inflammatory, lipidic and fibroblastic profile of epicardial stroma from patients who underwent open‐heart surgery, their regulation by cholinergic activity and its association with AF. We performed in vitro and ex vivo assays from paired subcutaneous and epicardial stromal cells or explants from 33 patients. Acute ACh effects in inflammation and lipid‐related genes were analysed by qPCR, in intracellular calcium mobilization were performed by Fluo‐4 AM staining and in neutrophil migration by trans‐well assays. Chronic ACh effects on lipid accumulation were visualized by AdipoRed. Plasma protein regulation by parasympathetic denervation was studied in vagotomized rats. Our results showed a higher pro‐inflammatory profile in epicardial regarding subcutaneous stromal cells. Acute ACh treatment up‐regulated monocyte chemoattractant protein 1 levels. Chronic ACh treatment improved lipid accumulation within epicardial stromal cells (60.50% [22.82‐85.13] vs 13.85% [6.17‐23.16], *P* < .001). Additionally, patients with AF had higher levels of fatty acid‐binding protein 4 (1.54 ± 0.01 vs 1.47 ± 0.01, *P = *.005). Its plasma levels were pronouncedly declined in vagotomized rats (2.02 ± 0.21 ng/mL vs 0.65 ± 0.23 ng/mL, *P* < .001). Our findings support the characterization of acute or chronic cholinergic activity on epicardial stroma and its association with AF.

## 1 INTRODUCTION

Atrial fibrillation (AF) prevalence varies from 1% to 3% worldwide. It is estimated to increase by 22% in elderly population.^1^ This arrhythmia is associated with long‐term mortality, morbidity and higher risk of stroke. The responsible factors for the AF incidence and perpetuation can be classified into different mechanisms, which give this disease a multi‐factorial condition.^2^, ^3^, ^4^ Several studies described obesity as one of the main risk factors of AF.^5^, ^6^, ^7^, ^8^ Bodyweight gain is associated with hypervolemia, left atrial enlargement, ventricle diastolic dysfunction and neurohormonal activation. All these mechanisms might explain some AF triggers.^5^ However, the advance in non‐invasive imaging techniques allowed the classification of different fat depots and pointed out the association of epicardial adipose tissue (EAT) with cardiovascular diseases.^9^ In particular, epicardial fat has been related with this arrhythmia, in an obesity‐independent manner.^10^, ^11^

Some authors described the contribution of EAT‐released fibrotic and inflammatory factors to the atrial structural tissue remodelling or AF substrate.^12^, ^13^, ^14^ Other observational studies suggested the disruption of electrical waves among cardiomyocytes when this fat tissue infiltrated into myocardium.^15^ These studies suggest a new direct role of this fat pad in AF development or progression.

As cardiac ganglionated plexus clusters are embedded in this fat pad,^6^, ^16^ it can also participate in the autonomic nervous system misbalance and, in consequence, in AF burden.^17^ In this sense, some treatments were focused on ganglionated plexuses ablation.^18^ Although they are constituted by parasympathetic and sympathetic elements, a recent clinical trial has demonstrated the reduction of long‐term atrial tachyarrhythmias in paroxysmal AF patients who underwent cardiac surgery, by specific parasympathetic denervation with botulinum toxin injection on EAT.^19^, ^20^, ^21^ However, the unsuccessful results achieved in some patients, as well as the association between EAT and AF recurrence after catheter ablation,^22^ suggest a possible interaction between parasympathetic activity and epicardial fat in arrhythmogenesis perpetuation. One of the most important parasympathetic neurotransmitters is acetylcholine (ACh), which can regulate the endocrine/paracrine activity of epicardial fat.^23^ The dialogue between mature adipocytes and the stromal vascular fraction (SVF), (fibroblasts, adipose‐derived or mesenchymal, vascular and immune cells) confers an important role on adipose tissue function and differentiation.^24^ The derangement of this communication might imply molecular and structural changes involved in cardiovascular arrhythmogenesis with unmet medical and therapeutic needs. Although several authors have focused their attention on the mechanisms underlying the effect of epicardial fat‐released factors on cardiomyocytes, we wanted to clarify the molecular changes in epicardial stroma after acute and chronic cholinergic activity and its association with AF.

## 2 MATERIALS AND METHODS

### 2.1 Human samples

Epicardial adipose tissue (EAT) of the right ventricle and paired subcutaneous adipose tissue (SAT) of the thoracic region were obtained from 33 patients who underwent open‐heart surgery. All patients have signed informed consent. The exclusion criteria were previous heart surgery or severe infectious diseases. Atrial fibrillation was considered as the permanent (chronic) form of the arrhythmia, which cannot be corrected by treatment. Galician Clinical Research Ethics Committee approved the study protocol, which was carried out in accordance with the Declaration of Helsinki. Before extra‐corporeal pulmonary circulation, small fat biopsies were cut with scissors. Instantaneously, the collected samples were placed in a phosphate buffered saline solution that contained the following (in mM): 0.5 EDTA, 5 KCl, 10 HEPES, 2 MgCl_2_, 10 NaHCO_3_, 0.5 KH_2_PO_4_, 0.5 NaH_2_PO_4_, 10 glucose, 110 NaCl and 0.16 CaCl_2_ (pH 7.4).

### 2.2 Animal design

For the murine experiments, we selected Sprague‐Dawley rats. Rats were housed in air‐conditioned rooms (22‐24°C) under controlled light/dark cycle (12 hours/12 hours) with food and water ad libitum (n = 16). The surgical procedure was performed aseptically, and with sterilized instruments. Surgeries were performed under anaesthesia by intraperitoneal injection of a mixture of ketamine/xylazine (100 mg/kg bodyweight + 15 mg/kg bodyweight, respectively). The rats were positioned on their backs, and a midline abdominal incision was made. The liver was carefully relocated to the right to expose the oesophagus. Dorsal and ventral branches of the vagus nerve were exposed and dissected. Each branch was ligated at two points with surgical sutures, as distally as possible to prevent bleeding, and cauterized between the sutures. The abdominal muscles and the skin were then closed with surgical silk (n = 8). Sham surgeries were also performed, in which each trunk of the nerve was exposed but not tied or cauterized (n = 8). The effectiveness of the vagotomy was evaluated by post‐mortem stomach observation. One week later, the animals were killed by exposure to an increasing concentration of CO_2_. Only the rats that presented an increase in stomach size after vagotomy were included.^25^ Upon decapitation, trunk blood was collected and immediately centrifuged, and plasma was stored at −80°C for the biochemical measurements.

The animal work in this study was approved by the Animal Care Committee of Santiago de Compostela University (Santiago de Compostela, Spain) in accordance with our institutional guidelines and the European Union standards for the care and use of experimental animals. The approaches in the present manuscript were performed under the procedure 15005/2015/003 reviewed and approved by the Faculty Animal Committee at the University of Santiago de Compostela.

### 2.3 Intracellular calcium mobilization in human SVC

We seeded the stromal vascular cells (SVC) from SAT and EAT of 3 patients (3 plates/fat pad) in 35 mm glass‐bottom culture dishes (MatTek Corporation), at ~40% confluence to avoid cell overlapping. Overlapping would obstruct the subsequent analysis. Prior to the experiment, SVC were serum‐deprived with M199 overnight. The day of the experiment, plates were washed twice with standard Tyrode's solution containing the following components (135 mM NaCl, 4 mM KCl, 10 mM glucose, 10 mM 2‐[4‐(2‐hydroxyethyl)piperazin‐1‐yl]ethanesulphonic acid (HEPES), 2 mM CaCl_2_ and 1 mM MgCl_2_ at pH 7.4). SVC were loaded in the same buffer with 2 µM of the calcium‐specific fluorescent dye Fluo‐4 AM from Invitrogen (Thermo) at 37°C with 5% CO_2_ for 1 hour. Once Fluo‐4 AM was internalized, and extracellular dye was removed twice with Tyrode's solution and post‐incubated at room temperature for 15 minutes to hydrolyse cytoplasmic Fluo‐4 AM. Dishes were mounted on the stage of Leica LS8 fluorescence inverted microscope (objective 45X) (Leica Biosystems). Fluorescent images were captured every 0.375 second at an excitation wavelength of 490 nm with an emission wavelength of 535 nm. After 30 seconds to establish a stable image baseline where it was possible, the response to vehicle (Tyrode's solution), acetylcholine (ACh) (Sigma) at 10 µM and ionomycin (Invitrogen) at 1 µM was determined (we waited for 60 seconds between each addition in order to allow cellular response). Calcium response was calculated using a ratio between ionomycin response (maximal Ca^+2^ level) and ACh or vehicle response. The maximum response after each addition and before the next condition was used to comparison analyses. Each analysis evaluated the response of 5‐10 representative whole cells per plate. Images were analysed with Fiji: ImageJ software.^26^

### 2.4 Inflammation, lipid and fibroblast‐related gene expression levels in SVC

After washing the fat pads three times, SAT and EAT SVC from 12 patients were isolated and cultured following the collagenase digestion protocol.^27^ Then, cells were or not induced to adipogenesis with M199 medium (Lonza Biologics) supplemented with 10% foetal bovine serum (FBS), and the adipogenesis cocktail, called IDMT, composed by 5 μg/mL insulin, 250 nM dexamethasone, 0.5 mM methylisobutylxanthine and 1 μM thiazolidinedione^28^ for 21 days. All pharmacological drugs were obtained from Sigma‐Aldrich and used at the same concentrations previously described,^29^ with minor modifications. At the end of the process, primary culture cells were lysed with AllPrep DNA/RNA/protein mini kit (Qiagen) and RNA was obtained, following the manufacture's protocol, for the level determination of the fibrosis, inflammation and lipid‐related genes selected below. After retro‐transcription, using the Maxima First Strand cDNA Synthesis Kit (Thermo Fisher Scientific), 2 μL of cDNA was used for CCAAT/enhancer‐binding protein beta (*C/EBPβ*), collagen type I alpha 2 (*COL1A2*), fatty acid‐binding protein 4 (*FABP4*), monocyte chemoattractant protein 1 (*MCP1*), perilipin A (*PLN A*), peroxisome proliferator‐activated receptor gamma (*PPARγ*), Interleukin 6 (*IL‐6*), pre‐adipocyte factor 1 (*PREF1*) and β‐actin (*ACTB*) amplification using the FastStart SYBR Green Master (Roche Diagnostics), and the primers detailed in Table S1. These primers were amplified at 40 cycles (95°C for 30 seconds, 60°C for 60 seconds) in a Stratagene Mx3005P (Agilent Technologies). The cycle threshold (Ct) values of the genes were normalized by the Ct values of ACTB (ΔCt). The differential expression levels were represented based on 2^−(ACTB/gene)^ algorithm.

### 2.5 Trans‐well assay for differentiated HL‐60 cells

ACh chemotaxis assay in differentiated HL‐60 (dHL‐60) was performed following the protocol previously described^30^ with minor modifications. Briefly, the human promyeloblast HL‐60 cell line (ATCC) was cultured and differentiated with 1.25% dimethylsulphoxide (DMSO) in RPMI with 10% FBS and penicillin/streptomycin for 7 days. We seeded 4 × 10^5^ dHL‐60 cells on each experimental condition. The concentrations selected for chemoattractants were 11 nM C5a as positive control, 10 µM ACh and conditioned medium from SAT and EAT SVC with or without pre‐treated with 10 µM ACh (n = 3). Cells were allowed to migrate for 2 hours at 37°C and 5% CO_2_. Neutrophils adhered to the bottom of the trans‐well were detached with 0.5 M ethylenediaminetetraacetic acid (EDTA) for 15 minutes at 4°C. The number of cells in the bottom of each well was manually counted using images taken at 4X with an inverted microscope Leica DMIL (Leica Biosystems) by two independent researchers. Images were analysed with ImageJ2 analysis program.^31^ Cell count average was calculated for each condition/experiment. Migration was measured as cell count ratio between each condition and control.

### 2.6 Fat tissue supernatants and plasma FABP4 protein levels

Fat pads from 10 patients were rinsed three times, dried and split into pieces of 100 mg weight. After washing overnight, samples were or not treated with 10 µM ACh (Sigma‐Aldrich) during 30′ at 37°C with 5% CO_2_. FABP4 concentrations were analysed with a Magnetic Luminex assay (R&D Systems), following the manufacturer's protocol. Briefly, 1:2 dilution was incubated with microparticles for 2 hours at room temperature on a shaker at 800 rpm. Then, biotin antibody was added for 1 hour and streptavidin‐PE for 30 minutes after washing between incubations. Fluorescence was detected within 90 minutes using a Bio‐Rad Bio‐Plex analyser (Bio‐Rad).

### 2.7 Lipid accumulation in SAT and EAT SVC

Primary culture of SVC from paired SAT and EAT of 5 patients were or not treated with ACh for 21 days, twice a week. At the end of treatment, lipid droplets, triglycerides accumulation, were visualized after 15 minutes of incubation with AdipoRed Assay Reagent (Lonza Biologics, SL) at room temperature. Representative images were captured with 20X objective of an inverted Axio Vert. A1 Zeiss Microscope (Carl Zeiss Microscopy GmbH). AdipoRed fluorescence intensity was recorded in a FluoStar Optima fluorimeter (BMG Labtech GmbH) and depicted as fluorescence relative units (RFU). The average of 6 independent wells was calculated per treatment and fat tissue from 5 independent patients.

### 2.8 Statistical analysis

Normal distributions were assessed by Shapiro‐Wilk test. Quantitative variables were presented either as mean ± standard error media (SEM) or as median (interquartile range) (IQR), according they were normally distributed or skewed data, respectively. Clinical continuous variables (normally distributed data) were presented as mean ± deviation standard, categorical variables were presented as frequency and percentage, and differences were analysed by chi‐squared Pearson test. Paired comparisons between treatments or fat pads were determined by Wilcoxon signed‐rank test or paired t test according to the normality of data. Differences between patients with respect to AF were defined by Mann‐Whitney signed‐rank test or unpaired t test according to the normality of data. Statistical significance was defined as *P* < .05. All analyses were performed using SPSS v22.0. (Software SPSS Inc).

## 3 RESULTS

### 3.1 Gene expression comparative between human SAT and EAT SVC

**FIGURE 1:**
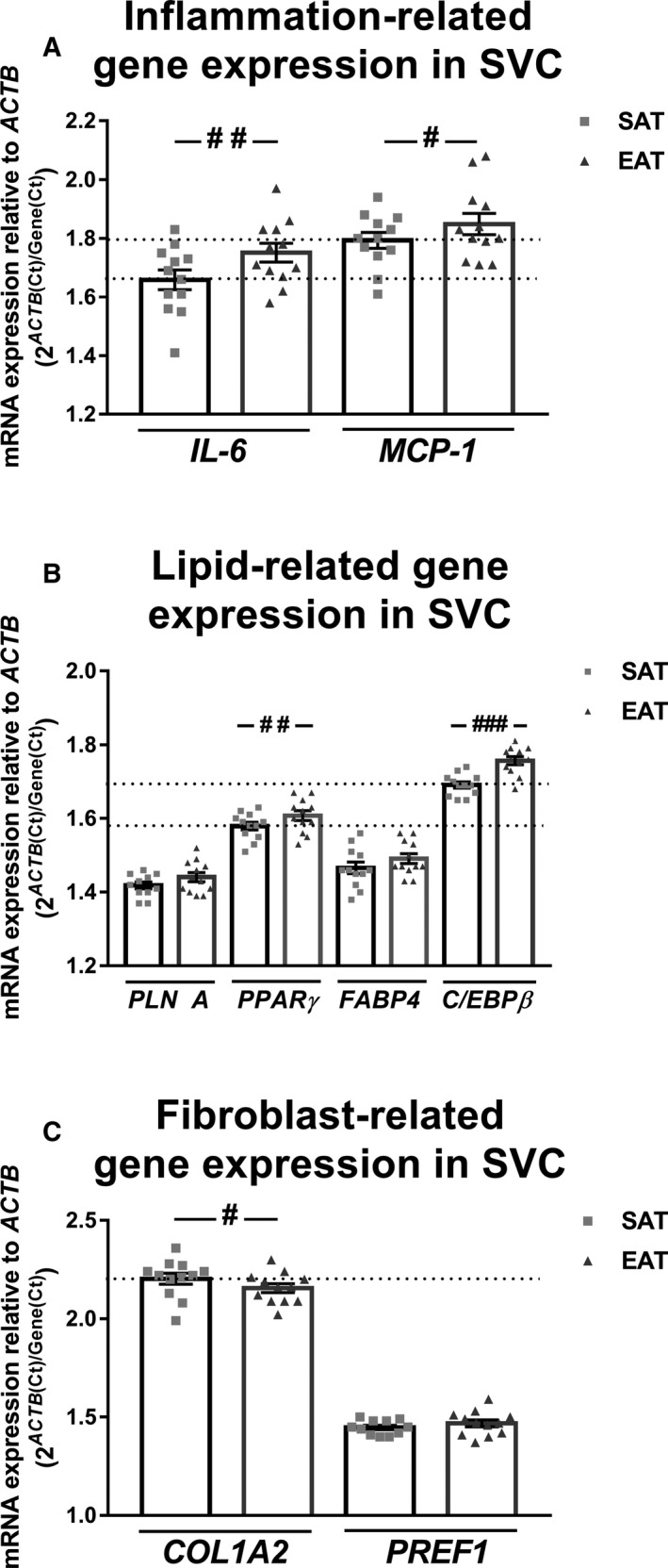
Gene expression comparative between human subcutaneous (SAT) and epicardial adipose tissue (EAT) stromal vascular cells (SVC). Interleukin 6 (*IL‐6*) and monocyte chemoattractant protein 1 (*MCP1*) (A); perilipin 1 (*PLINA*), peroxisome proliferator‐activated receptor gamma (*PPARγ*), fatty binding protein 4 (*FABP4*) and CCAAT/enhancer‐binding protein beta (*C/EBPβ*) (B); and collagen type I alpha II (*COL1A2*) and pre‐adipocyte factor 1 (*PREF1*) (C) mRNA expression levels relative to actin (*ACTB*) in EAT or SAT SVC (n = 12). Dot plots with bar depict individual values and mean ± SEM. Differences between tissues were evaluated by paired t test and represented with a line and pounds. Significant values are expressed as ^#^ *P* < .05 and ^##^ *P* < .01

We performed an analysis of the *IL‐6* and *MCP1* inflammation‐related gene mRNA expression levels in paired SAT and EAT SVC from 12 patients (69 ± 9 years old, body mass index (BMI): 28.75 ± 3.84 kg/m^2^, 50% with coronary artery disease (CAD), 16.7% with type 2 diabetes mellitus (T2DM), 66.7% hypertensive, 8.3% with heart failure (HF) and 33.3% with AF). EAT showed an elevated inflammatory profile regarding to SAT SVC (*IL‐6*:1.77 ± 0.03 vs 1.66 ± 0.03, *P* = .002 and *MCP1*: 1.85 ± 0.04 vs 1.79 ± 0.03, *P = *.013) (Figure 1A).

Our results showed higher *PPARγ* expression levels in EAT SVC than in SAT SVC (1.61 ± 0.01 vs 1.58 ± 0.01, *P = *.007). On the same line, *C/EBPβ* mRNA expression was greater in EAT SVC in comparison with SAT SVC (1.76 ± 0.01 vs 1.69 ± 0.008, *P* < .0001) (Figure 1B).

On the contrary, collagen I alpha II (*COL1A2)* expression levels were lower in EAT with respect to SAT SVC (2.16 ± 0.02 vs 2.20 ± 0.03, *P = *.018) (Figure 1C).

### 3.2 ACh effect on intracellular calcium mobilization from human SAT and EAT SVC

**FIGURE 2:**
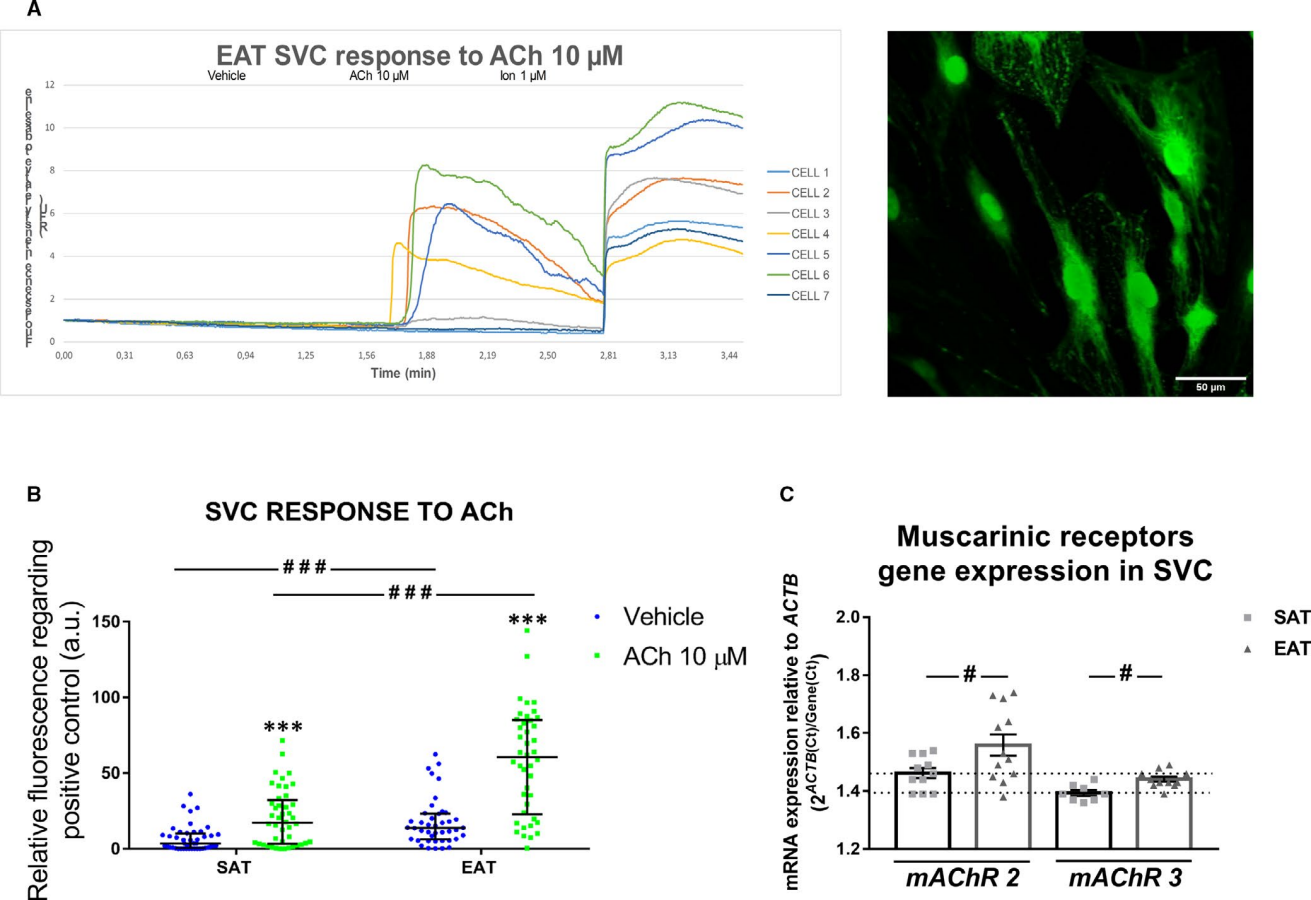
Intracellular calcium mobilization by ACh treatment in stromal vascular cells (SVC) from human subcutaneous (SAT) and epicardial (EAT) adipose tissues. Representative case showing the time‐course fluorescence quantification of 7 cells from one plate of EAT SVC, performed with Fiji: ImageJ software On the right side, the superposition of all the frames analysed in the video of this case to allow the characterization of the cells (objective 45X) (A). Comparison of ACh fluorescence intensity between SAT and EAT SVC from 3 patients (5‐10cells/3 plates/tissue) was performed by Wilcoxon ranked test and represented with asterisks. Dot plots with bar depict individual values and median [Interquartile range (IQR)] (B). Differences between fat pads are represented with lines and pounds and depicted as ^###^ *P* < .001. Differences between vehicle and treatment are expressed as *P < .001. mRNA expression levels of muscarinic receptors *mAChR2* and *mACR3* in EAT SVC and SAT SVC are represented by dot plots with bar (C). Differences between fat pads are represented with lines and pounds and depicted as ^#^ *P* < .05

ACh effect in stromal cells was validated by testing intracellular calcium mobilization. This cholinergic neurotransmitter can trigger a myriad of responses in different cell types apart from the classic neuronal autonomic pathway.^32^, ^33^, ^34^ We wanted to test whether SVC were able to respond to this neurotransmitter in samples from 3 patients (77 ± 4 years old, body mass index (BMI): 29 ± 3 kg/m^2^, 33% with coronary artery disease (CAD), 33% with type 2 diabetes mellitus (T2DM), 100% hypertensive and 66% with AF). Ionomycin was selected as positive control, representing the maximum intracellular calcium level by fluorescence intensity (Figure 2A). We selected a 10 µM Ach concentration according our previous publication, confirming that it is an effective concentration in its endocrine activity.^23^ As can be seen in Figure 2B, ACh at 10 µM induced calcium mobilization in 60% of EAT SVC cells and 61% of SAT SVC. This response was greater in EAT SVC (60.50% [22.82‐85.13] vs 13.85% [6.17‐23.16], *P* < .001), than in SAT SVC (17.18% [3.37‐32.31] vs 3.58% [0.44‐10.09], *P* < .001), regarding vehicle treatment. After analysing the mRNA expression levels of muscarinic receptors *mAChR2* (Gi protein‐coupled receptor) sand *mAChR3* (Gs protein‐coupled receptor), we observed higher expression levels in EAT SVC than in SAT SVC (1.56 ± 0.13 vs 1.46 ± 0.058 for *mAChR2* and 1.44 ± 0.03 vs 1.39 ± 0.03 for *mAChR3*; *P* < .05) (Figure 2C).

### 3.3 Gene expression regulation of human SAT and EAT SVC and adipogenesis‐induced SAT and EAT SVC under Ach treatment

**FIGURE 3:**
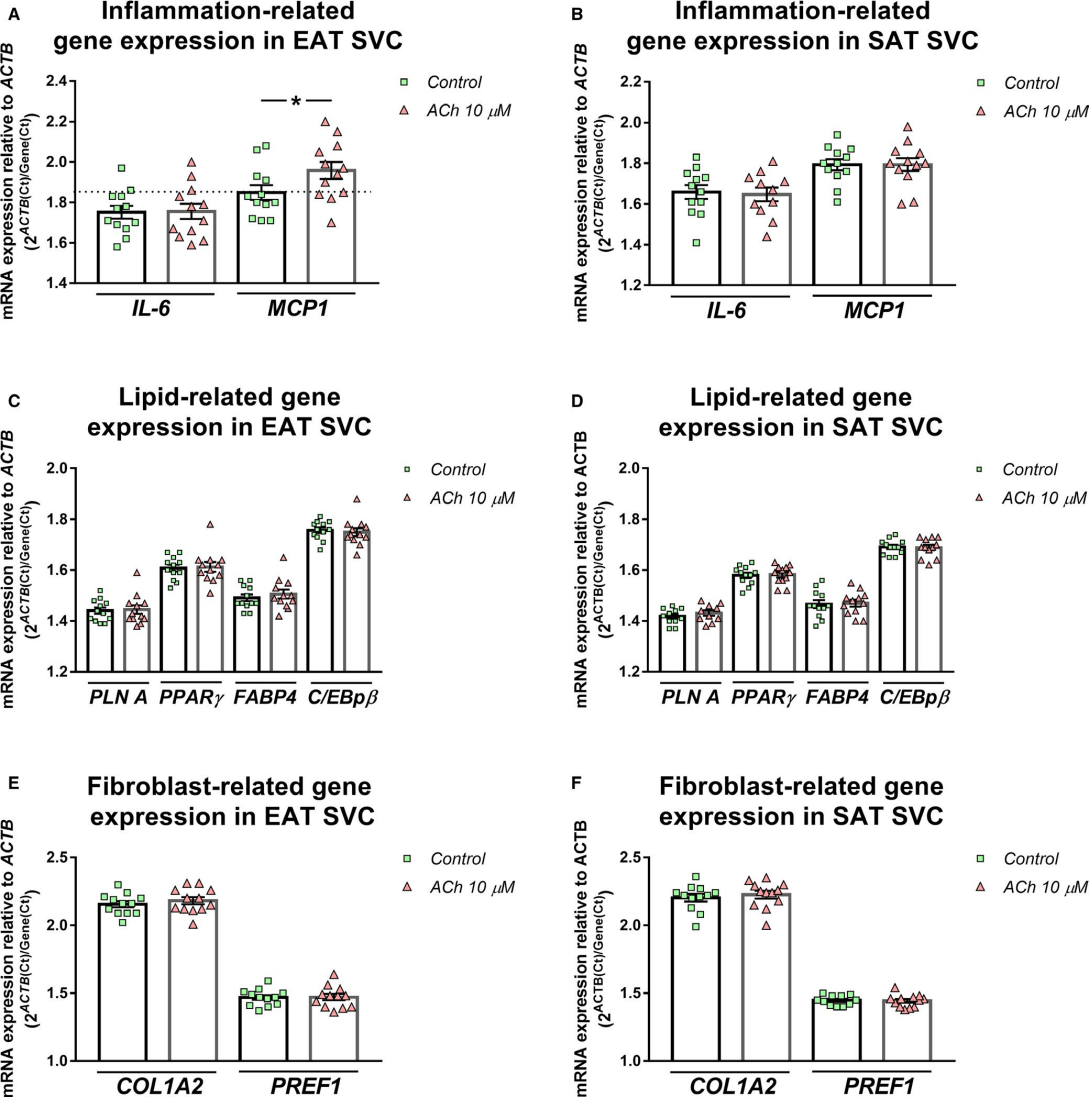
Inflammation, lipid and fibroblast‐related gene regulation by acute ACh treatment in human epicardial stromal vascular cells (SVC). Interleukin 6 (*IL‐6*) and monocyte chemoattractant protein 1 (*MCP1*) (A, D); perilipin 1 (*PLINA*), peroxisome proliferator‐activated receptor gamma (*PPARγ*), fatty binding protein 4 (*FABP4*) and CCAAT/enhancer‐binding protein beta (*C/EBPβ*) (B, E); and collagen type I alpha II (*COL1A2*) and pre‐adipocyte factor 1 (*PREF1*) (C, F) mRNA expression levels relative to actin (*ACTB*) in epicardial SVC (A, B, C) or subcutaneous SVC (D, E, F) (n = 12). Dot plots with bar depict individual values and mean ± SEM. Differences between vehicle and treatment were evaluated by paired t test and represented with a line and asterisks. Significant values are expressed as *P < .05

**FIGURE 4:**
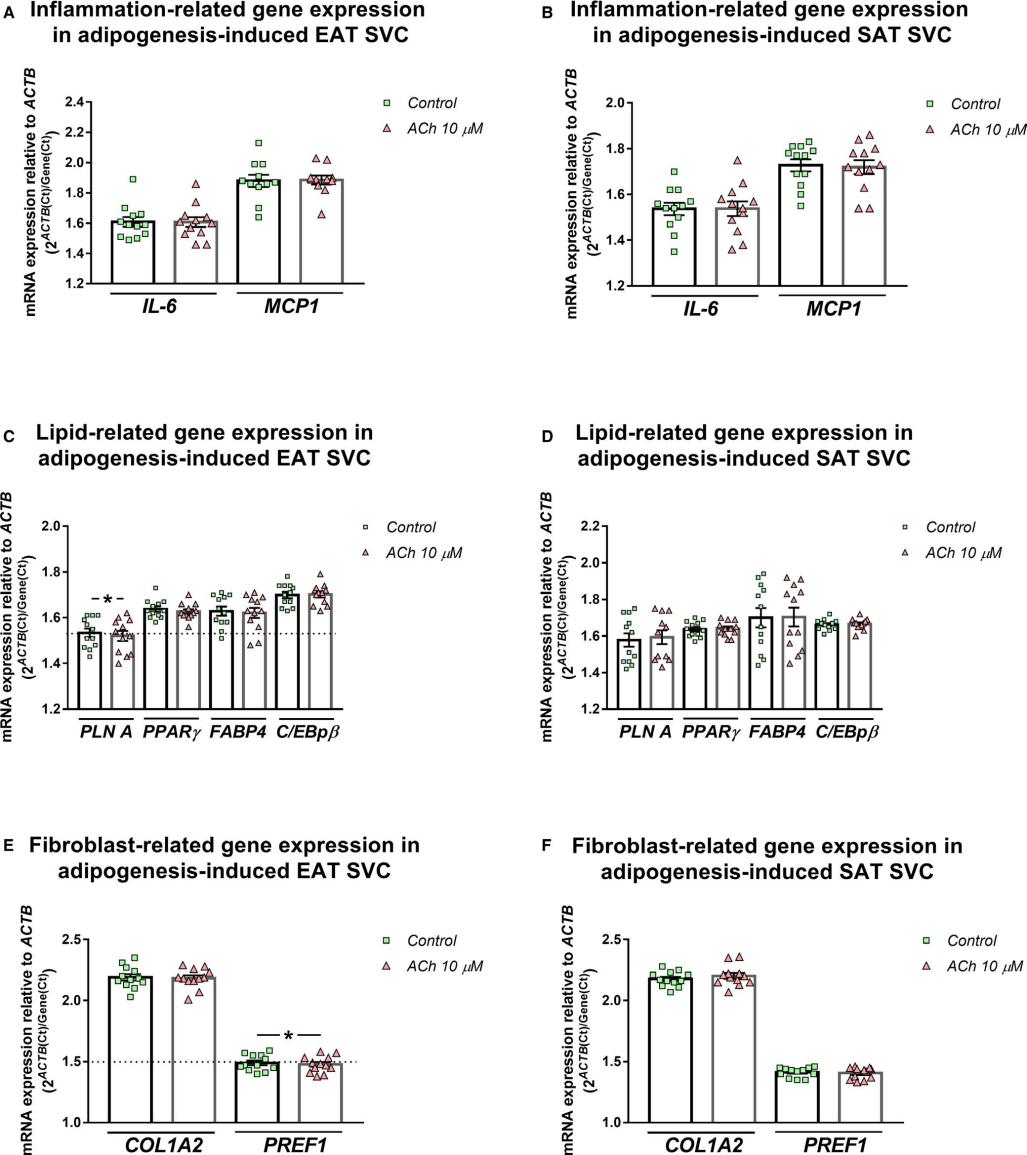
Inflammation, lipid and fibroblast‐related gene regulation by acute ACh treatment in human subcutaneous and epicardial adipogenesis‐induced stromal vascular cells (SVC). Interleukin 6 (*IL‐6*) and monocyte chemoattractant protein 1 (*MCP1*) (A, D); perilipin 1 (*PLINA*), peroxisome proliferator‐activated receptor gamma (*PPARγ*), fatty binding protein 4 (*FABP4*) and CCAAT/enhancer‐binding protein beta (*C/EBPβ*) (B, E); and collagen type I alpha II (*COL1A2*) and pre‐adipocyte factor 1 (*PREF1*) (C, F) mRNA expression levels relative to actin (*ACTB*) in epicardial SVC (A, B, C) or subcutaneous SVC (D, E, F) (n = 12). Dot plots with bar depict individual values and mean ± SEM. Differences between vehicle and treatment were evaluated by paired t test and represented with a line and asterisks. Significant values are expressed as *P < .05

Once we knew that SVC responded to ACh, we tested the previously studied genes. Acute ACh treatment (30 minutes) up‐regulated *MCP1* gene expression levels in EAT SVC (1.96 ± 0.04 vs 1.85 ± 0.04, *P = *.023) (Figure 3A). We did not see any differences regarding other genes (Figure 3B,C) neither when we analysed SAT SVC (Figure 3D‐F). In addition, gene expression levels were tested after adipogenesis differentiation induction. At this time, acute ACh treatment did not modify the *MCP1* or *IL‐6* expression levels (Figure 4A). *PLN A* mRNA expression was slightly down‐regulated after ACh treatment (1.51 ± 0.02 vs 1.53 ± 0.02 control, *P* < .022) (Figure 4B). *PREF1* results were in the same line (1.48 ± 0.02) vs control (1.49 ± 0.02, *P* < .043) (Figure 4C). As it was seen in non‐differentiated cells, adipogenesis‐induced SAT SVC showed no differences after ACh treatment (Figure 4D‐F).

### 3.4 Cholinergic activity promoted neutrophil migration and lipid accumulation in human SVC

**FIGURE 5:**
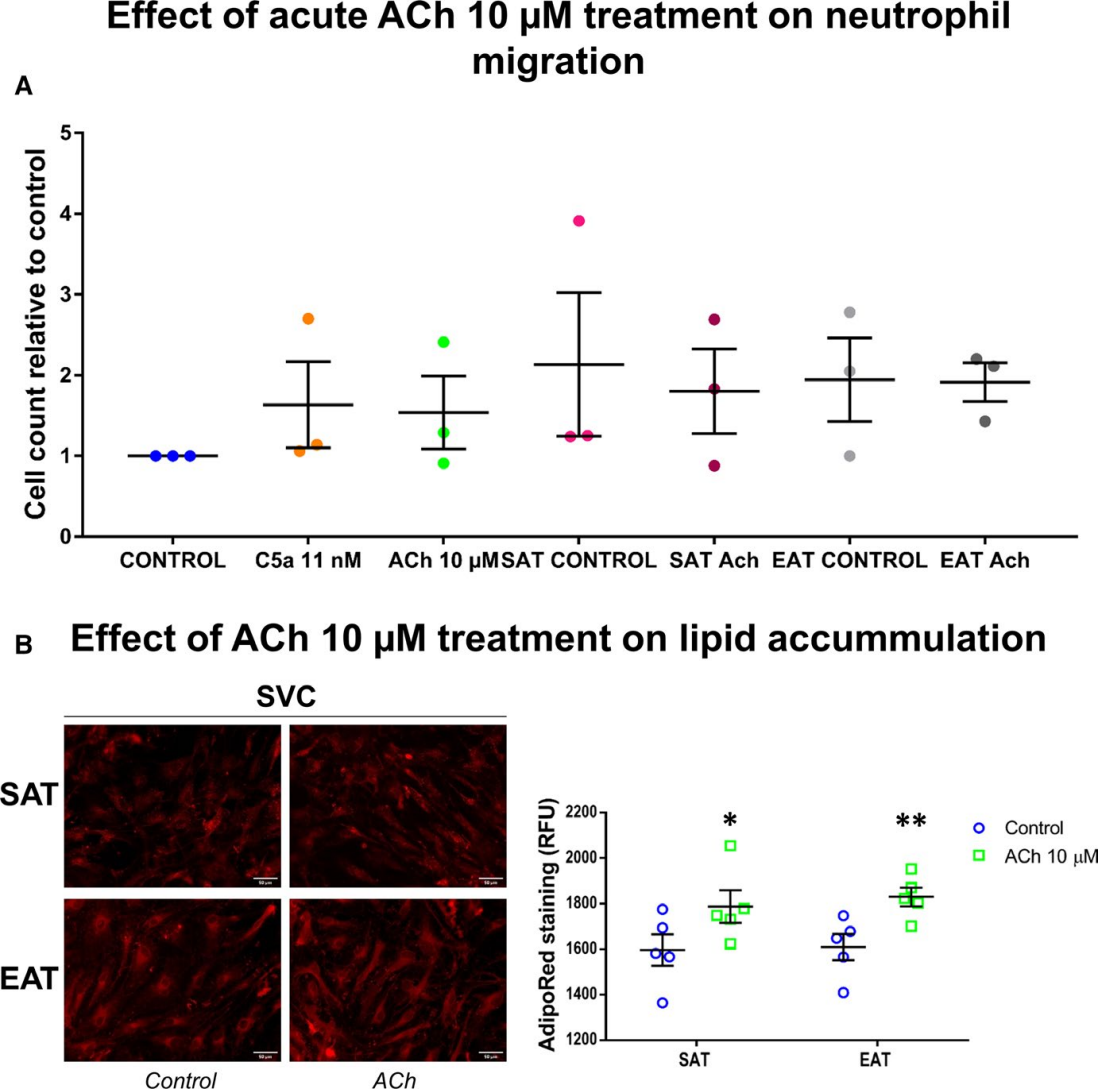
Neutrophil migration by acute ACh treatment and lipid accumulation by chronic ACh treatment. Migrated cells count relative to basal control were analysed after acetylcholine (ACh) or conditioned medium from ACh treated or not ‐subcutaneous and epicardial stromal vascular. Complement component 5 alpha (C5a) was used as positive control (A). Representative images showing AdipoRed lipid staining in SVC from subcutaneous (SAT) and epicardial (EAT) adipose tissue after ACh treatment for 21 days. AdipoRed Assay was quantified by fluorimetry from 5 independent patients (n = 5). Dot plots depict individual values and mean ± SEM. Comparisons between control and treatment were analysed by paired t tests. Significant values represent *P < .05 and **P < .01 (B)

After differentiating HL‐60 promyelocytic cell line into neutrophils, we analysed ACh effect in their migration ability. Our results showed a trend to increase neutrophil migration after ACh treatment (1.54 ± 0.45) over positive control, C5a. Conditioned medium from SAT and EAT SVC improved this process (2.13 ± 0.89 in SAT and 1.94 ± 0.52 in EAT) (n = 3) (Figure 5A). However, the variability of the assay did not allow to reach statistical significances.

As we wanted to test ACh effect in lipid accumulation, we performed a chronic treatment (21 days, ACh 10 µM added with the media twice a week) in primary stromal cells from 5 patients without AF (75 ± 9 years old, body mass index (BMI): 30.5 ± 1.28 kg/m^2^, 60% with coronary artery disease (CAD), 40% with type 2 diabetes mellitus (T2DM), 80% hypertensive, 40% with heart failure (HF)). Intriguingly, we observed an increase in intracellular lipids content in both SAT (1787.63 ± 71.77 vs 1596.00 ± 69.49 RFU, *P* < .05) and EAT SVC fluorescence intensities (1829.93 ± 41.37 vs 1609.47 ± 57.93 RFU, *P* < .01) in comparison with their controls (n = 5) (Figure 5B).

### 3.5 FABP4 gene expression differences between patients with or without permanent atrial fibrillation and its parasympathetic regulation in vagus nerve‐denervated rats

**FIGURE 6:**
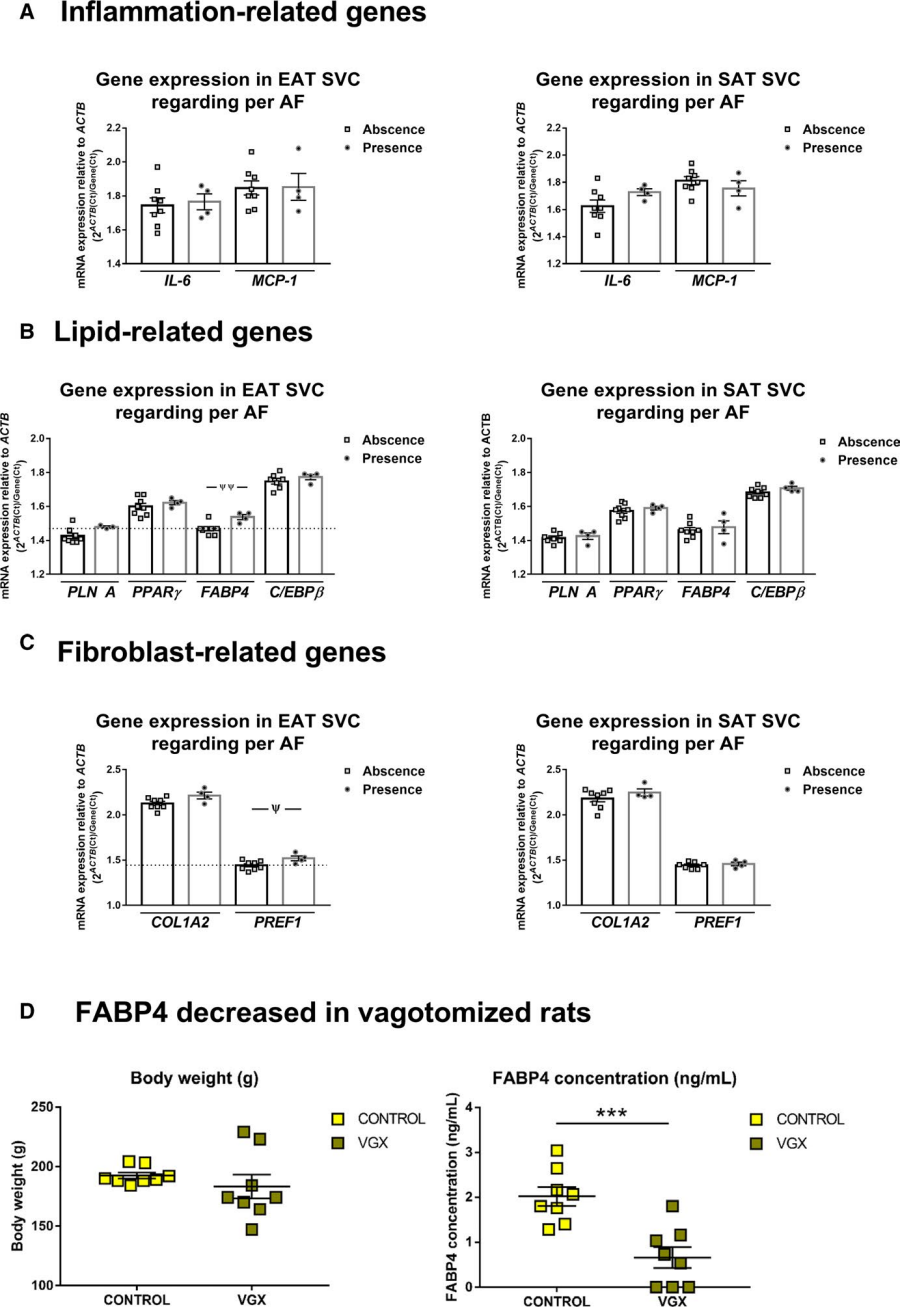
Differential expression of genes in patients regarding permanent atrial fibrillation (AF) and plasma levels regulation by cholinergic denervation. Interleukin 6 (*IL‐6*) and monocyte chemoattractant protein 1 (*MCP1*) (A, D); perilipin 1 (*PLINA*), peroxisome proliferator‐activated receptor gamma (*PPARγ*), fatty binding protein 4 (*FABP4*) and CCAAT/enhancer‐binding protein beta (*C/EBPβ*) (B, E); and collagen type I alpha II (*COL1A2*) and pre‐adipocyte factor 1 (*PREF1*) (C, F) mRNA expression levels relative to actin (*ACTB*) in epicardial SVC (A, B, C) or subcutaneous SVC (D, E, F) (n = 12). Comparison between patients with or without permanent AF was analysed by unpaired *t* tests and depicted as ^¥^ *P* < .05 and ^¥¥^ *P* < .01. Plasma FABP4 levels and bodyweight were determined or recorded in rats with or without vagus nerve denervation for seven days (n = 16). Whisker plots depict mean ± SEM. Comparison among subjects was performed with unpaired t test. Significant values represent ***P < .001 (D)

Inflammatory genes did not differ regarding AF (Figure 6A). However, *FABP4* levels were up‐regulated (1.54 ± 0.01 vs 1.47 ± 0.01, *P = *.005) (Figure 6B) and *PREF1* mRNA expression levels in EAT SVC from AF patients (1.52 ± 0.03 vs 1.44 ± 0.01, *P = *.020) (Figure 6C). As can be seen in Table 1, clinical characteristics did not differ among patients regarding AF. Any change was detected in SAT SVC regarding AF (Figure 6A‐C), After analysing FABP4 secretion by EAT explants from 10 patients (75 ± 7 years old, BMI: 27.87 ± 3.30 kg/m^2^, 20% with CAD, 30% with T2DM, 70% hypertensive, 50% with HF and 40% with AF), we observed a similar secretion levels between EAT and SAT (Figure S1). However, its parasympathetic long‐term regulation was demonstrated in a vagotomized rat model Plasma FABP4 levels were reduced after vagus nerve denervation (0.65 ± 0.23 ng/mL vs 2.02 ± 0.21 ng/mL, *P < *.001), without bodyweight changes (183.10 ± 10.09 g vs 192.30 ± 2.58 g) (n = 16) (Figure 6D).

**TABLE 1:** Clinical characteristics of the study population of SVC according atrial fibrillation (AF) presence

Variables	Non‐AF patients n = 8	AF patients n = 4	*P*‐value
Age (years), (mean ± SD)	66 ± 11	74 ± 2	.368
Gender (male), n (%)	6 (75)	4 (100)	.273
BMI (kg/m^2^), (mean ± SD)	28.13 ± 4.26	30.00 ± 3.00	.368
T2DM, n (%)	1 (12.5)	1 (25)	.584
CAD, n (%)	5 (62.5)	1 (25)	.221
HF, n (%)	1 (12.5)	0 (0)	.460
Hypertension, n (%)	6 (75)	2 (50)	.386
LVEF < 50%, n (%)	1 (12.5)	1 (25)	.584
VR surgery, n (%)	2 (25)	2 (50)	.433
CABG surgery, n (%)	3 (37.5)	0 (0)	.188

Abbreviations: BMI, body mass index; CABG, coronary artery bypass graft; CAD, coronary artery disease; HF, heart failure; LVEF, ejection fraction; T2DM, type II diabetes mellitus; VR, valve replacement.

## 4 DISCUSSION

For the first time, our results demonstrated an acute and chronic ACh effect on epicardial stromal cells. This neurotransmitter up‐regulated the inflammatory response and lipidic accumulation in a time‐course depending manner. Whereas acute ACh treatment increased *MCP1* and calcium mobilization, chronic treatment enhanced the lipidic accumulation. In this regard, patients with permanent AF had higher levels of the lipid marker *FABP4* in epicardial stroma than those without AF. Its chronic regulation by sympathetic/parasympathetic disbalance caused in vagotomized rat models, and the stronger association between plasma FABP4 levels with atrial fat tissue in persistent regarding to paroxysmal AF,^35^ suggest the important role of the cholinergic activity effect on epicardial fat development. Although most of the studies tried to understand the crosstalk between epicardial fat and myocardial cells, the new results suggest the adrenergic/cholinergic disbalance as regulator of epicardial fat development and activity.

Autonomic nervous system is composed by ganglionated plexus clusters, which are distributed over the myocardium and embedded in epicardial fat pad. Denervation of the cholinergic system in this pad by botulinum toxin injection (which prevents the ACh release) has shown a considerably reduction of AF after coronary artery bypass surgery.^19^, ^20^, ^21^ This study suggests the modulation of AF triggers embedded into epicardial fat as a new preventive strategy. However, cholinergic activity might also activate mechanisms on epicardial stromal cells through muscarinic receptors^23^ with consequences on epicardial fat development and activity. We analysed the ACh effect on stromal cells through intracellular calcium assays because its role on signalling pathways.^36^ Besides, it induces fast responses that facilitate its use as a positive control. The higher expression levels of muscarinic receptors types 2 and 3 in epicardial stromal cells might explain their pronounced response regarding subcutaneous stromal cells.

The relationship between obesity and low‐grade systemic inflammation is well known. The latter has shown to be involved in AF onset and persistence.^3^, ^37^, ^38^, ^39^, ^40^ In this study, we also demonstrated that specifically epicardial fat stroma has a greater pro‐inflammatory profile (as can be seen by *MCP1* and *IL‐6* gene expression levels) than SAT. Acute cholinergic activity increased the MCP1 expression in epicardial stroma. Thus, the reduction of cholinergic activity on epicardial fat during open‐heart surgery might prevent the acute inflammatory response and neutrophils infiltration, similar mechanism to colchicine treatment, which it was used as preventive strategy for post‐operative AF.^41^ The expression levels of adipogenesis‐related transcription factors, *PPARγ* and *C/EBP*β, were higher in subcutaneous than epicardial stromal cells. Thus, the subcutaneous stromal cells might get a faster differentiation and lipidic response than epicardial stromal cells.

In fact, acute ACh treatment slightly down‐regulated the expression levels of *PLN A,* a lipid droplet‐associated protein, and *PREF1*, pre‐adipocyte factor 1 (absent in mature adipocytes). The down‐regulation of *PREF1* might suggest an induction of adipocyte differentiation, as it was described in fibroblast‐myofibroblasts differentiation.^42^ However, this mechanism is not clear when perilipin expression levels were also down‐regulated. The confuse response might suggest the need of a chronic cholinergic treatment for testing the lipidic effects on stromal cells. Thus, we performed assays with chronic ACh treatment to clarify this process for 21 days. Our results showed an enhance of lipid accumulation in SAT and EAT SVC. Our previous findings showed an association of both local and peripheral FABP4 (a carrier protein for fatty acids) concentration levels with left atrial adipose tissue volume in persistent but not in paroxysmal AF patients.^35^ Additionally, our results confirmed that epicardial stroma has already a higher lipidic profile in permanent AF. Taking all our results together, we get to one hypothesis that goes in line with the findings obtained in murine models by Furuhashi et al^43^ In fact, the long‐term implication of the cholinergic system in FABP4 regulation was evidenced when these protein levels were sharply decreased in vagus nerve‐denervated rats. Although further studies are needed to clarify the mechanism, our findings suggest that cholinergic activity could promote lipid accumulation with higher fatty acids transport into the epicardial stroma. This molecule might co‐ordinate the pro‐inflammatory and metabolic activity in stromal vascular cells and macrophages, taking part in AF progression. Modulation of the cholinergic activity might reduce the pro‐inflammatory profile and long‐term lipid accumulation of epicardial stroma and, in consequence, AF development or progression.

### 4.1 Limitations

This is a single‐centre, small‐sample population study. Due to ethical reasons, healthy EAT samples are unavailable. We do not perform heart transplant neither enough autopsies to get EAT from cardiovascular healthy donors. As it comes from the same patient (allowing us to discard subject‐related confounders), we selected paired SAT samples for identifying specific responses and changes on EAT. We performed studies in ventricle adipose tissue because of the results that pointed out the similarities between atrial and ventricle adipose tissue regarding ACh response, and it is safer to obtain it.^22^ In addition, atrial and ventricle ganglionated plexi are interconnected and cooperate as a single unit. The regulation of one of them will affect the other.^41^ Intracellular calcium mobilization analyses were performed in adherent SVC in different plates. Direct injection on a single cell might improve time and dose courses under ACh treatment. Long‐term vagus nerve denervation effect on plasma FABP4 was performed in a rat's model.

## 5 CONCLUSIONS

Acute ACh activity up‐regulates *MCP1* chemokine expression levels and calcium mobilization on epicardial stromal cells. Longer ACh treatment enhanced lipid accumulation in this fat pad. In this line, epicardial stroma from patients with permanent AF contains higher FABP4 expression levels. Thus, modulation of cholinergic activity might reduce FABP4 as vagus nerve denervation is associated with a sharply decrease in FABP4 plasma levels.

## CONFLICT OF INTEREST

None declared.

## AUTHORS' CONTRIBUTIONS

MCS, JN.LC, ARL and SEP had made substantial contributions to the conception and design of this study, and the analysis and interpretation of data. ALF, DDM, LMS and LGM were responsible for the acquisition of samples. MRM, JR.GJ, MCS and SEP had been involved in drafting the manuscript and revising it critically for important intellectual content. All authors have given final approval of the version to be published. Each author had participated sufficiently in this work to take public responsibility for appropriate portions of the content and agreed to be accountable for all aspects of the work in ensuring that questions related to the accuracy or integrity of any part of it are appropriately investigated and resolved.

## Supporting information

Fig S1

Table S1

## Data Availability

The data that support the findings of this study are available on request from the corresponding author. The data are not publicly available due to privacy or ethical restrictions.
