# Supplementary figures and images for: Inflammatory and lipid regulation by cholinergic activity in epicardial stromal cells from patients who underwent open‐heart surgery
Source: J Cell Mol Med. 2020 Aug 7;24(18):10958–69. doi: 10.1111/jcmm.15727 (PMC7521153; doi:10.1111/jcmm.15727)

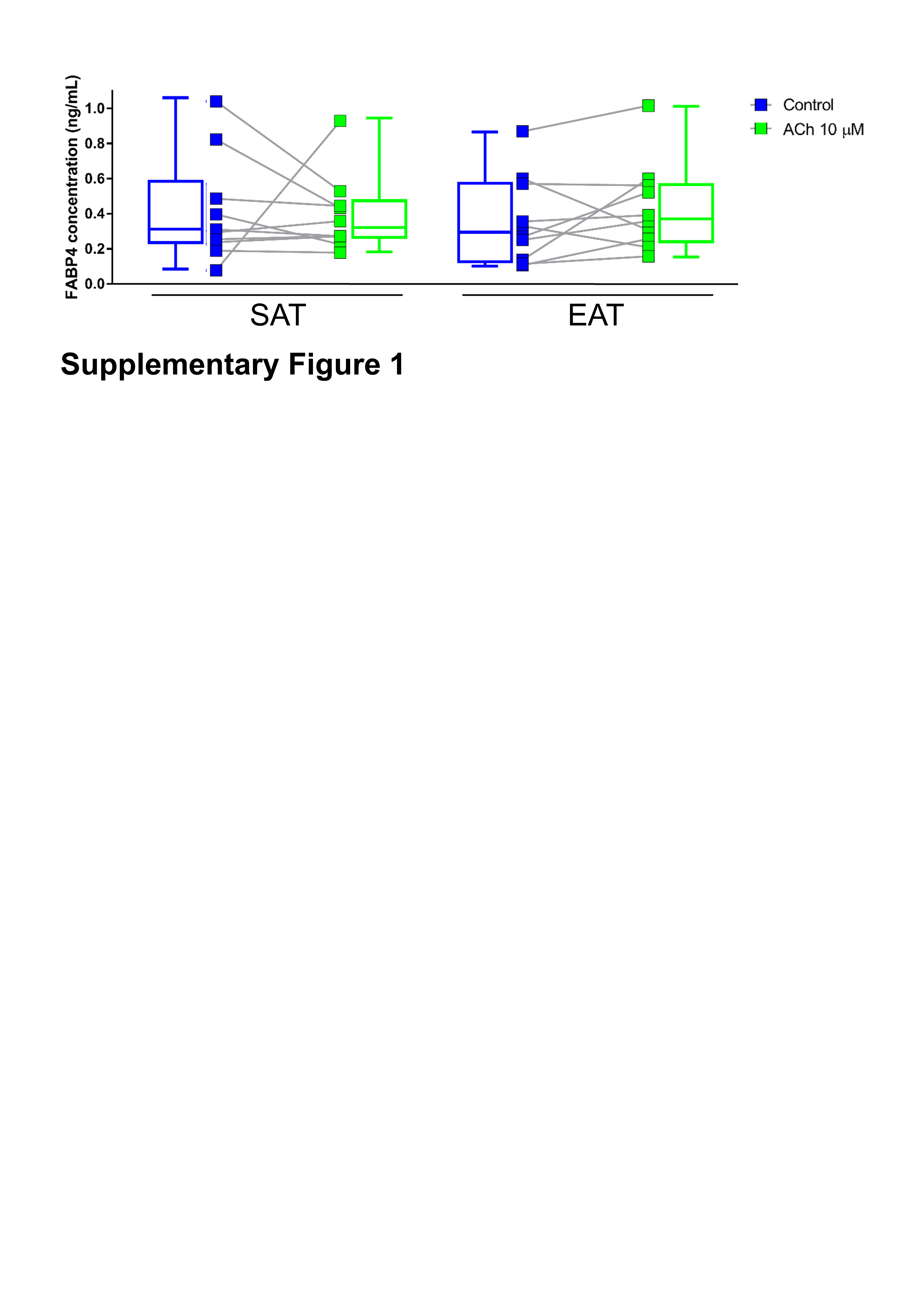

Supplement: Supplementary file 1 — Fig S1 [file JCMM-24-10958-s001.tif]
