# Supplementary material for: Inflammatory and lipid regulation by cholinergic activity in epicardial stromal cells from patients who underwent open‐heart surgery
Source: J Cell Mol Med. 2020 Aug 7;24(18):10958–69. doi: 10.1111/jcmm.15727 (PMC7521153; doi:10.1111/jcmm.15727)
Supplement: Supplementary file 2 — Table S1 [file JCMM-24-10958-s002.docx]

**Supplementary Table 1**

Primer sequences of human genes used in Real Time PCR.

| Gene | Forward primer | | Reverse primer | | | *Reference* |
| --- | --- | --- | --- | --- | --- | --- |
|  | |  | |  |  |  |
| ***ACTB*** | ttctgacccatgcccaccat | | atggatgatgatatcgccgcgctc | | | ^1^ |
| ***C/EBPβ*** | CACAGCGACGACTGCAAGATCC | | CTTGAACAAGTTCCGCAGGGTG | | | 2 |
| ***COL1A2*** | TCGCACATGCCGTGACTTG | | GATAGCATCCATAGTGCATCCTTG | | | ^3^ |
| ***FABP4*** | TACTGGGCCAGGAATTTGAC | | GTGGAAGTGACGCCTTTCAT | | | ^1^ |
| ***IL-6*** | tgaggtgcccatgctacattt | | gtggctgcaggacatgacaa | | | ^4^ |
| ***MCP1*** | caaactgaagctcgcactctc | | gctgcagattcttgggttgtg | | | ^5^ |
| ***mAChR2*** | CTCCAGCCATTCTCTTCTGG | | GCAACAGGCTCCTTCTTGTC | | | ^6^ |
| ***mAChR3*** | CGCTCCAACAGGAGGAAGTA | | GGAGTTGAGGATGGTGCTGT | | | ^7^ |
| ***PLN A*** | CTCTCGATACACGTGCAGA | | TGGTCCTCATGATCCTCCTC | | | ^8^ |
| ***PPARγ*** | TGTCTCATAATGCCATCAGGTTTG | | GATAACGAATGGTGATTTGTCTGTT | | | ^9^ |
| *PREF1* | CTGGACGGTGGCCTCTATGAATG | | ATCATCCACGCAGGTGCCTC | | | ^3^ |

ACTB: β actin, C/EBPβ: CCAAT/enhancer-binding protein beta, COL1A2: Collagen type I α2, FABP4: Fatty acid binding protein 4, IL-6: Interleukin 6, MCP1: Monocyte quemo-attractant protein 1, PLN A: perilipin A, PPARγ: peroxisome proliferator activated-receptor γ, PREF1: preadipocyte factor 1

1. Agra, R. M., Fernández-Trasancos, Á., Sierra, J., González-Juanatey, J. R. & Eiras, S. Differential Association of S100A9, an Inflammatory Marker, and p53, a Cell Cycle Marker, Expression with Epicardial Adipocyte Size in Patients with Cardiovascular Disease. *Inflammation* **37**, 1504–1512 (2014).
2. Yang H Park J Woo D Jeon S Do H Lim H Kim J Park K. C/EBP-α and C/EBP-β-mediated adipogenesis of human mesenchymal stem cells (hMSCs) using PLGA nanoparticles complexed with poly(ethyleneimmine) Biomaterials 2011 vol: 32 (25) pp: 5924-5933
3. Abdallah, B. M. *et al.* Regulation of human skeletal stem cells differentiation by Dlk1/Pref-1. *J. Bone Miner. Res.* **19**, 841–852 (2004).
4. Eiras, S. et al. Extension of coronary artery disease is associated with increased IL-6 and decreased adiponectin gene expression in epicardial adipose tissue. Cytokine 43, 174–180 (2008).
5. Eiras, S. *et al.* Relationship between epicardial adipose tissue adipocyte size and MCP-1 expression. *Cytokine* **51**, 207–212 (2010).
6. Ferretti M, Fabbiano C, Di Bari M, et al. M2 receptor activation inhibits cell cycle progression and survival in human glioblastoma cells. *J Cell Mol Med*. 2013;17(4):552-566. doi:10.1111/jcmm.12038
7. Pacini L, De Falco E, Di Bari M, et al. M2muscarinic receptors inhibit cell proliferation and migration in urothelial bladder cancer cells. *Cancer Biol Ther*. 2014;15(11):1489-1498. doi:10.4161/15384047.2014.955740
8. Hah, Y. S. *et al.* Cultured human periosteal-derived cells have inducible adipogenic activity and can also differentiate into osteoblasts in a perioxisome proliferator-activated receptor-mediated fashion. *Int. J. Med. Sci.* **11**, 1116–1128 (2014).
